# Microarchitecture of the tsetse fly proboscis

**DOI:** 10.1186/s13071-017-2367-2

**Published:** 2017-09-19

**Authors:** Wendy Gibson, Lori Peacock, Rachel Hutchinson

**Affiliations:** 1 School of Biological Sciences, University of Bristol, Bristol, BS8 1TQ UK; 2 School of Clinical Veterinary Science, University of Bristol, Langford, Bristol, BS40 7DU UK

**Keywords:** *Glossina*, Tsetse, *Trypanosoma congolense*, Proboscis, Hypopharynx, Labrum, Labium, Labellum, Blood-sucking, Haematophagous

## Abstract

**Background:**

Tsetse flies (genus *Glossina*) are large blood-sucking dipteran flies that are important as vectors of human and animal trypanosomiasis in sub-Saharan Africa. Tsetse anatomy has been well described, including detailed accounts of the functional anatomy of the proboscis for piercing host skin and sucking up blood. The proboscis also serves as the developmental site for the infective metacyclic stages of several species of pathogenic livestock trypanosomes that are inoculated into the host with fly saliva. To understand the physical environment in which these trypanosomes develop, we have re-examined the microarchitecture of the tsetse proboscis.

**Results:**

We examined proboscises from male and female flies of *Glossina pallidipes* using light microscopy and scanning electron microscopy (SEM). Each proboscis was removed from the fly head and either examined intact or dissected into the three constituent components: Labrum, labium and hypopharynx. Our light and SEM images reaffirm earlier observations that the tsetse proboscis is a formidably armed weapon, well-adapted for piercing skin, and provide comparative data for *G. pallidipes*. In addition, the images reveal that the hypopharynx, the narrow tube that delivers saliva to the wound site, ends in a remarkably ornate and complex structure with around ten finger-like projections, each adorned with sucker-like protrusions, contradicting previous descriptions that show a simple, bevelled end like a hypodermic needle. The function of the finger-like projections is speculative; they appear to be flexible and may serve to protect the hypopharynx from influx of blood or microorganisms, or control the flow of saliva. Proboscises were examined after colonisation by *Trypanosoma congolense* savannah. Consistent with the idea that colonisation commences in the region nearest the foregut, the highest densities of trypanosomes were found in the region of the labrum proximal to the bulb, although high densities were also found in other regions of the labrum. Trypanosomes were visible through the thin wall of the hypopharynx by both light microscopy and SEM.

**Conclusions:**

We highlight the remarkable architecture of the tsetse proboscis, in particular the intricate structure of the distal end of the hypopharynx. Further work is needed to elucidate the function of this intriguing structure.

**Electronic supplementary material:**

The online version of this article (10.1186/s13071-017-2367-2) contains supplementary material, which is available to authorized users.

## Background

In tropical Africa tsetse flies (genus *Glossina*) are the vectors of sleeping sickness (human African trypanosomiasis, HAT) and the livestock disease nagana or African animal trypanosomiasis (AAT). There are 23 species of tsetse varying in size from 6 to 16 mm in length [1, 2]. In contrast to other dipteran vectors, both male and female tsetse feed on blood [3], taking a new blood meal every few days during the several weeks of their lifespan. Different species vary in their preferred hosts, but most will feed on a range of large vertebrates, including humans. Typical natural hosts are large mammals such as buffalo, antelope and wild suids, or large reptiles such as crocodiles and monitor lizards, but some species favour elephants and rhino, leastways they did in the past before these animals became rare [4, 5].

Feeding from this range of hosts necessitates mouthparts strong enough to pierce thick skin and detailed drawings and microscopic images of the tsetse proboscis reveal a formidable, well-armed organ with arrays of sharp teeth and rasps [6–8]. These structures are carried on the paired labella that form the tip of the proboscis and are normally held inverted within the proboscis until the fly feeds; after the proboscis penetrates the skin, the labella are repeatedly everted and inverted, enabling the rasps and teeth to tear into the skin tissue [7]. Eversion of the labella is controlled partly by muscles located in the bulb of the proboscis and partly by haemostatic pressure [6, 7]. Blood pools in the wound and is mixed with saliva, issuing from the hypopharynx, as it is sucked up the food canal, with suction force provided by the cibarial pump, located at the back of the tsetse head [1, 6].

The proboscis also serves as the developmental site for trypanosomes of several species of livestock trypanosomes, including the major pathogens *Trypanosoma congolense* and *T. vivax*. Epimastigotes multiply in the proboscis attached to the walls of the food canal and cibarial pump [9–13], and subsequently invade the hypopharynx and become infective metacyclics that are inoculated into the host with fly saliva [14]. While *T. vivax* completes its whole developmental cycle in the proboscis [9, 14], *T. congolense* initially multiplies in the fly midgut before invading the proboscis via the proventriculus and foregut [14, 15]. Relatively few studies of the development of these trypanosomes in the proboscis have been carried out and many questions remain. For example, how the diverse morphological stages observed link into the life-cycle [15], how the trypanosomes locate their developmental sites and what metabolic substrates support the growth of large populations of trypanosomes.

To gain a better understanding of the physical environment the proboscis offers for trypanosome development, we have re-examined the microarchitecture of the proboscis of both trypanosome infected and non-infected *Glossina pallidipes* using light and scanning electron microscopy (SEM). As previous studies have focussed mainly on *Glossina palpalis* and *G. morsitans*, our study provides comparative data on *G. pallidipes*.

## Methods

### Trypanosomes and tsetse

*Trypanosoma congolense* savannah strains Gam2, WG81, S104 and 1/148 were grown as procyclics in Cunningham’s medium (CM) [16] supplemented with 5 μg/ml hemin and 15% *v*/v heat-inactivated foetal calf serum at 27 °C. Tsetse flies (*Glossina pallidipes*) were kept in single sex groups of 10–20 per cage at 25 °C and 70% relative humidity, and fed on sterile defibrinated horse blood via a silicone membrane. Male or female flies were given an infected blood meal at their first feed 1–5 days after emergence. The infective blood meal consisted of procyclic trypanosomes (approximately 10^7^ cells/ml) in CM mixed with an equal volume of washed horse red blood cells resuspended in Hank’s Balanced Salt Solution, supplemented with 10 mM L-glutathione to increase infection rates [17].

### Dissection and light microscopy

Flies were cold-anaesthetized before removal of the head with a scalpel blade. The proboscis was separated into its three component parts, labrum, labium and hypopharynx, using forceps and hypodermic needles. Specimens to be observed by light microscopy were placed into a drop of phosphate buffered saline (PBS), covered with a coverslip and viewed by phase contrast. Infected proboscises for SEM were placed in PBS plus 20% foetal calf serum in a 24 well plate before fixation.

### Scanning electron microscopy

For environmental SEM, proboscises were dissected, placed directly on stubs and viewed using a Zeiss Evo 15LS scanning electron microscope. Other proboscises, including those infected with *T. congolense*, were prepared by fixation in 2.5% glutaraldehyde in 100 mM phosphate buffer (pH 7.4), followed by dehydration through an ethanol series and critical-point drying. Samples were then placed on a stub and sputter coated with gold/palladium. These samples were viewed either on a Zeiss Evo 15LS or FEI Quanta 200 field emission SEM.

### Statistical analysis

Chi-square test was used to evaluate differences between microarchitecture of the hypopharynx and sex of fly and proboscis infection.

## Results and discussion

### Microarchitecture of the proboscis tip

**Fig. 1:**
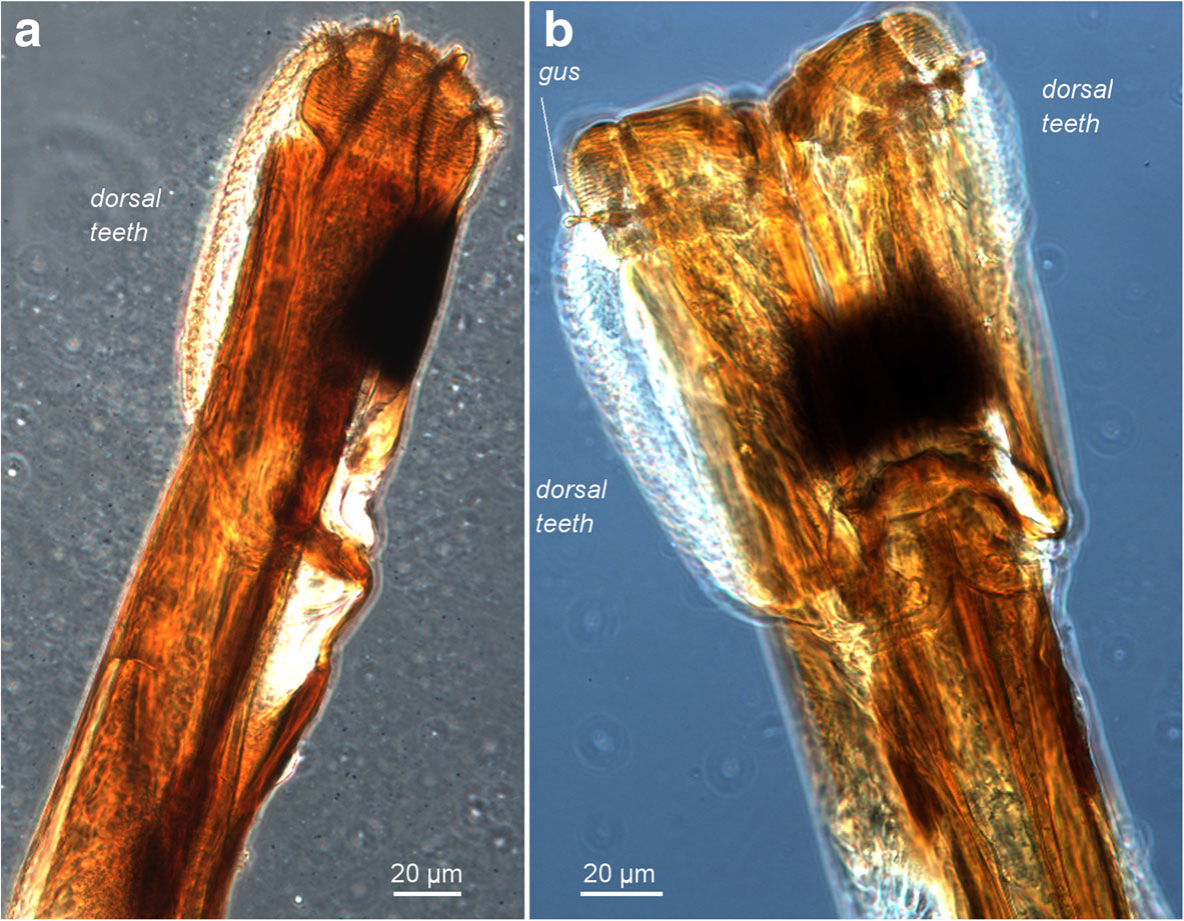
Skin piercing machinery - light microscopy. The tip of the *Glossina pallidipes* proboscis showing the arrays of rasps and teeth used to penetrate through the skin to find blood. Light microscopy images of the tip of the proboscis, inverted (**a**) and everted (**b**). The dark pigmented region is on the ventral side of the labella and there is a long array of teeth on the dorsal side. These dorsal teeth are on the left in the lateral view shown in **a**; the rasps near the tip of the proboscis are clearly visible through the labellum wall, with gustatory sensilla and denticles protruding slightly from the tip. In **b** the everted labella are seen in ventral view, with dorsal teeth visible on both sides. Both sets of rasps, each subdivided into three, are visible, and now the gustatory sensilla (*gus*) and prostomal teeth are exposed in a ring at the base of the rasps

**Fig. 2:**
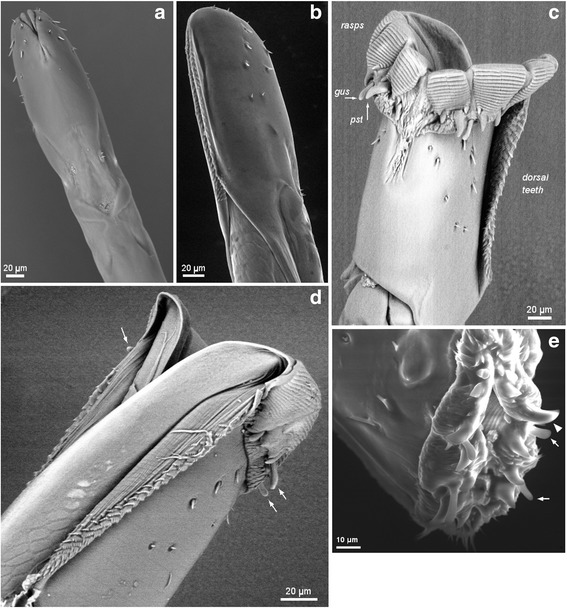
Skin piercing machinery - scanning electron microscopy. The tip of the *Glossina pallidipes* proboscis showing the arrays of rasps and teeth used to penetrate through the skin. Scanning electron microscopy images of the tip of the proboscis, inverted (**a, b**), everted (**c, d**) and partially everted (**e**). **a** Ventral view showing the small array of ventral teeth where the two labella meet centrally. **b** Dorso-lateral view showing the dorsal teeth in the groove formed by the closely opposed labella. **c** Everted labella in ventral view. The sets of rasps, each subdivided into three, are prominent; the small array of ventral teeth can be seen where the two labella meet centrally, as well as one of the larger arrays of dorsal teeth on the right-hand labellum. The gustatory sensilla (*gus*) and prestomal teeth (*pst*) are exposed at the base of the rasps. **d** Everted labella in dorsal view revealing the split between the labella on the dorsal side. Rasps and dorsal teeth are visible, and three of the eight gustatory sensilla are indicated (arrows). **e** Partially everted labella; ventral surface is at the top. Rasps visible within; two gustatory sensilla (arrows) and one prostomal tooth (arrowhead) are indicated

**Fig. 3:**
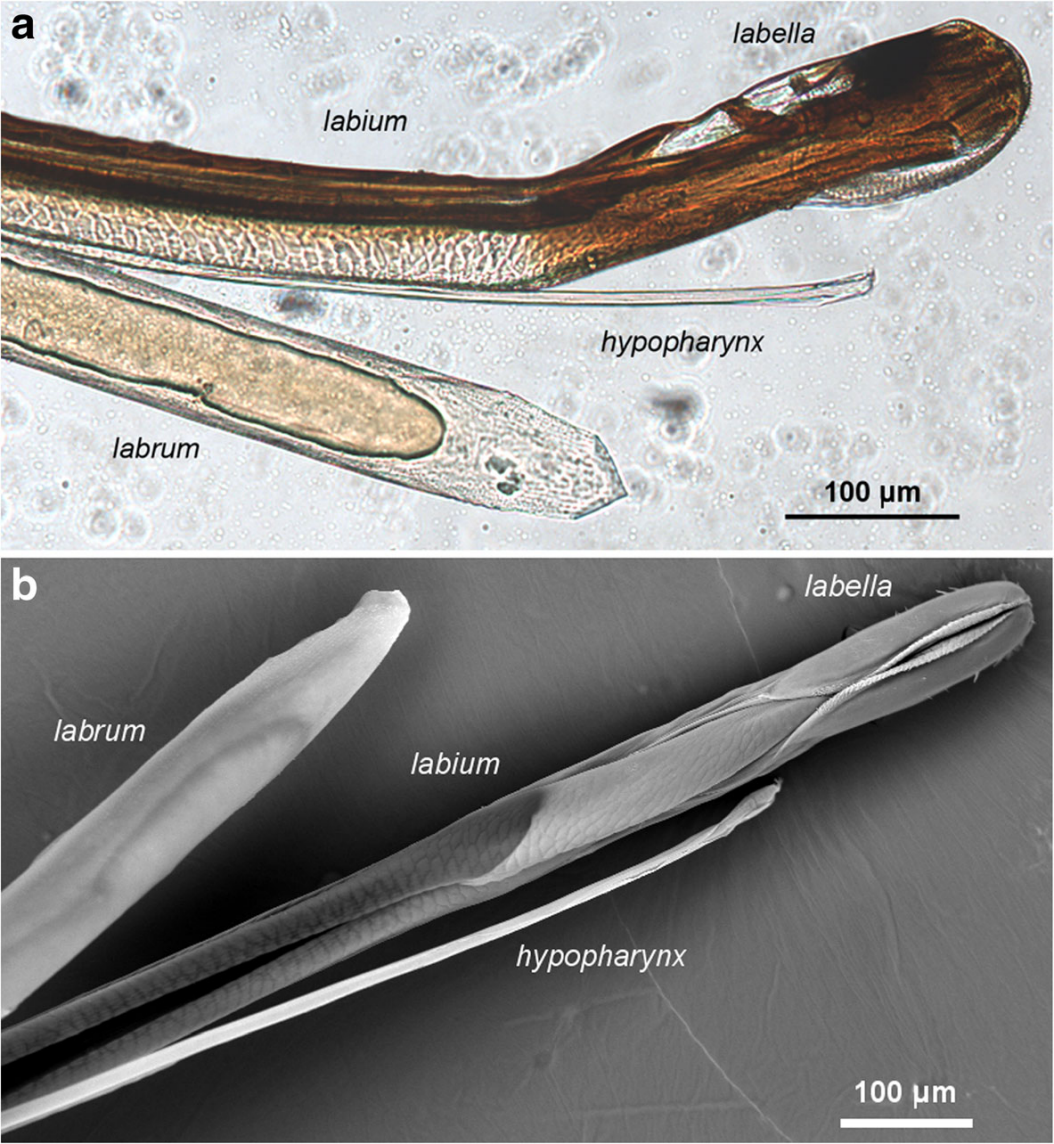
Component parts of the proboscis. The proboscis of *Glossina pallidipes* separated into its three component parts: labrum, labium and hypopharynx. **a** Light microscopy. **b** SEM, dorsal view of labium. The labrum interlocks with the labium approximately 400 μm from its distal tip, so that a continuous food canal is formed. The tip of the hypopharynx lies beyond the tip of the labrum, but does not reach the tip of the proboscis formed by the labella

**Fig. 4:**
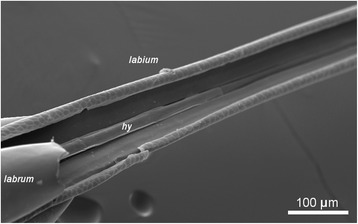
Labial groove. SEM image of proboscis with part of labrum removed showing the position of the hypopharynx (*hy*) within the groove in the labium. The lateral flanges of the hypopharynx can be clearly seen

The tsetse proboscis is normally held horizontally in front of the head and protected between two palps until the fly wants to feed, when it is lowered 90°, hinging between the head and proboscis bulb. The paired labella form the tip of the proboscis and these carry arrays of rasps and teeth for tearing through the skin, as well as sensory receptors (Figs. 1 and 2). The skin is pierced by the inverted labella, followed by repeated eversion and inversion of the armoured plates to tear the skin tissue and release blood, which is then mixed with anti-coagulant saliva delivered from the distal tip of the hypopharynx [7]. The blood is sucked up via the food canal, which is formed by the paired labella at the distal tip, and proximally by the labium (ventral) and labrum (dorsal), which zip together to form a continuous hollow tube. The relative positions of the three parts of the proboscis after dissection are shown in Fig. 3. During dissection, the hypopharynx can be teased gently away from the labium using a needle, but it is fragile and easily broken. In life, the hypopharynx lies in a groove in the labium (Fig. 4), except for the distal tip, which lies in the food canal between the two labella [1, 6].

### Microarchitecture of the hypopharynx

**Fig. 5:**
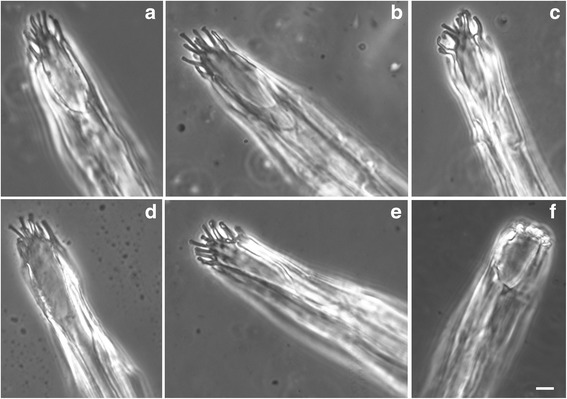
Distal tip of the hypopharynx. Structure of the hypopharynx tip. **a**-**f** Distal tip of hypopharynx by light microscopy. The hypopharynx ends in approximately ten finger-like projections of variable length. In **f**, the fingers are not obvious and appear to be retracted or curled up; this was the appearance in about one third of specimens examined. The opening of the salivary duct can be seen clearly in **a**. *Scale-bars*: 2 μm

In tsetse the hypopharynx is a narrow tube that carries saliva via the salivary duct from the paired salivary glands that lie in the thorax and abdomen [6]. In *G. pallidipes* the hypopharynx has a diameter of approximately 10 μm, with flattened regions or flanges of about 4 μm width on the sides (Fig. 4). In the literature, the hypopharynx is shown to end in a sharply pointed tip with an oblique opening, similar to the bevelled tip of a hypodermic needle [1, 6] and we were therefore surprised to observe that the tip of the hypopharynx appeared to have several finger-like projections in freshly-dissected specimens (Fig. 5 and Additional file 1: Movie 1). This tattered appearance did not result from mechanical damage, as it was observed in about two thirds of the flies dissected (present in 135 of 198 flies dissected), with no significant difference between male and female flies (*χ* ^2^ = 0.680, *df* = 1, *P* = 0.445). Nor was it a result of trypanosome infection, as there was no association with trypanosome infection (*χ* ^2^ = 0.740, *df* = 1, *P* = 0.439). The number and length of the finger-like projections varied between specimens (Fig. 5); generally about ten “fingers” could be counted. The apparent absence in about a third of specimens was probably the result of the “fingers” being folded or retracted in some way (Fig. 5f), as flies were matched for other variables such as age and environment.

**Fig. 6:**
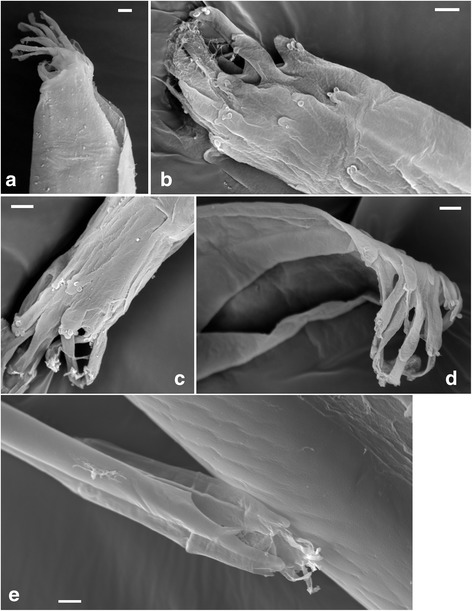
Microarchitecture of the hypopharynx tip. SEM images of the distal tip of the hypopharynx showing the complex structure of the finger-like projections. Five different specimens are shown (**a**-**e**). The opening of the salivary duct can be seen clearly in **d**. In **e** the tip of the hypopharynx is resting on the labium; each lateral flange ends in a broad tip. *Scale-bars*: **a**-**d**, 2 μm; **e**, 5 μm

We used scanning electron microscopy (SEM) to examine the structure of the hypopharynx tip in detail. Initial attempts to examine unfixed material were unsuccessful, because the fine structure was quickly lost as specimens dried out. Fixed and sputter-coated specimens gave excellent resolution, although the tip was still easily distorted and damaged by the electron beam unless supported by adherence to the stub. Figure 6 shows five different examples of the hypopharynx tip. Confirming the light microscopy, there appear to be at least ten finger-like projections, which vary in length from 3 to 8 μm and are approximately 1.2–1.5 μm in width. On the tips of some are small sucker-like protrusions. The dorsal views (Fig. 6b, c) show some rather short, rudimentary forms, sprouting from the exterior wall of the hypopharynx and also decorated with a few sucker-like protrusions, suggesting that the “fingers” grow longer with time, perhaps replacing damaged ones. Turnover through wear and tear would explain why a variable rather than constant number of fingers were observed in different flies. The prominent lateral flanges towards the tip of the hypopharynx terminate in blunt, rounded “thumbs”, distinct from the more delicate “fingers” (Fig. 6e).

**Fig. 7:**
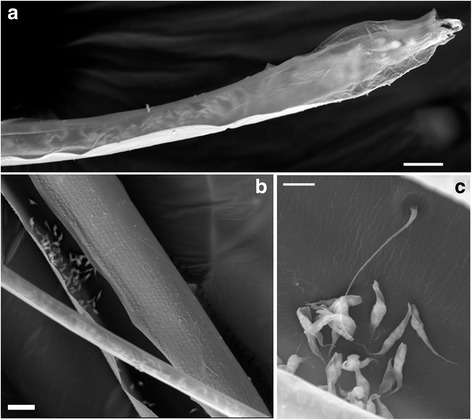
Trypanosome-infected proboscises. SEM images of tsetse mouthparts infected with *Trypanosoma congolense* savannah. **a** Distal end of hypopharynx. Trypanosomes can be seen densely packed inside the lumen. **b** Part of hypopharynx overlaying the labrum. Trypanosomes are visible on the internal wall of the labrum, and also as silhouettes within the hypopharynx. **c** Cluster of trypanosomes attached to the wall of the labrum close to a hair-like mechanoreceptor. *Scale-bars*: **a**, 10 μm; **b**, 20 μm; **c**, 5 μm

As the hypopharynx is actually a double-walled tube with the salivary duct forming the inner tube [6], it is possible that the “fingers” represent the tip of the salivary duct rather than the hypopharynx. While Figs. 6e and 7a could be interpreted in that way, in other images (e.g. Fig. 6b, c) the “fingers” undoubtedly arise from the exterior wall of the hypopharynx.

Observation of the hypopharynx tip in situ was not possible because it is covered by the thick, pigmented labella. Nevertheless, from observation of the orientation of the hypopharynx in dissected specimens, we deduce that the opening of the hypopharynx is downward-facing, such that the fingers curl down over the open tip, as in Fig. 6d. This concords with Jobling’s observation that the opening of the hypopharynx is ventral [6]. As the tip of the hypopharynx lies in the food canal between the two labella, when these separate dorsally during eversion (Fig. 2d), the tip of the hypopharynx is exposed, allowing saliva to flow directly into the pooled blood before it is imbibed [6].

The intricate structure of the tip of the hypopharynx revealed by SEM presupposes some purpose. The hypopharynges of other blood-sucking Diptera, such as midges, sand flies and blackflies, are part of the skin-piercing apparatus and as a consequence are heavily sclerotised and bear teeth or spines on the distal tip [3, 18–21]; however, in tsetse the heavily armoured labella do the work of piercing the skin, while the delicate hypopharynx is protected inside the labium. Similarly in mosquitoes, the two maxillae bear saw-like teeth, while the hypopharynx is a needle-like stylet without ornament [22, 23]. With no precedent in other blood-sucking insects, we can only speculate about the function of the tip of the tsetse hypopharynx.

The finger-like projections may serve to close the end of the salivary duct, perhaps to defend against entry of microorganisms or to regulate the flow of saliva, or may be sensory organs. The protective function hypothesis is weak, since trypanosomes, and presumably other microbes, are readily able to enter (and exit) the hypopharynx (Additional file 2: Movie 2 and Additional file 3: Movie 3). The regulation of salivary flow is a possibility, considering that questions remain on how the fly controls the release of saliva [1]. The salivary glands themselves are muscular, so that contractions push saliva through the narrower salivary ducts and hypopharynx. According to Jobling [6], backflow of saliva is prevented by closure of a muscular valve situated where the paired salivary ducts join together into the common salivary duct, but Buxton [1] queried whether the valve is strong enough to resist the pressure. Controlling the closure of the open tip of the hypopharynx might be an additional mechanism for regulating saliva flow. Closing the hypopharynx may also be useful for tsetse adapted to drier environments such as *G. pallidipes*. In this regard, it would be interesting to re-examine the hypopharynx of *G. palpalis*, a species adapted to more humid environments. Jobling’s detailed and meticulous drawings of the mouthparts of *G. palpalis* show the hypopharynx with a smooth, bevelled tip like that of a hypodermic needle [1, 6], but it is inexplicable that he missed this feature, unless it was not visible on the preserved material he used [1].

The position of the tip of the hypopharynx so close to the pool of blood as it is mixed with saliva during fly feeding might also suggest a sensory role for the “fingers” either in tasting the blood or monitoring its consistency. However, they do not resemble other mechano- and chemo receptors inside the labrum and labella (Figs. 2c, d, 7c), or indeed in other blood-sucking insects. Tsetse have gustatory sensilla on the labella (Fig. 2c-e), which are better placed to taste the host blood and indeed have been shown to detect the phago-stimulatory molecule, ATP [24]. Tsetse do discriminate between hosts and demonstrate learning of host preference [25], presumably based to some extent on sensory information from the proboscis.

### Trypanosome infection in proboscis

In dissecting flies to look for infection in the proboscis, it is generally easier to see trypanosomes in the labrum (Additional file 4: Movie 4), because the labium is thicker and more heavily pigmented. Indeed, early investigators commented that “The labium need not be examined as trypanosomes are never found in it if the labrum is negative” [26]. Trypanosomes appear to be able to attach to the walls of both the labrum and labium [13], but we have never found them attached to the outer surface of the hypopharynx by light microscopy or SEM (Fig. 7, Additional files 2, 3, 5: Movies 2, 3, 5), although the dorsal wall is an available surface for attachment in the food canal (Fig. 4). As reported previously [11, 27–29], trypanosomes are found attached near tsetse mechanoreceptors (Fig. 7c), impairing their function and interfering with feeding behaviour, such that infected flies probe more frequently; this favours trypanosome transmission and hence has profound implications for the epidemiology of the tsetse-transmitted trypanosomiases [27].

As *T. congolense* invades the proboscis from the proventriculus and foregut, it is logical that the proximal regions are the first places to be colonised by attached trypanosomes and this is indeed what is observed [13, 30, 31]. Our results confirm this generalization, in that the highest density of trypanosomes was found in the region of the labrum proximal to the bulb in about half the flies dissected (Table 1), but trypanosomes were also found in high density in the other regions of the labrum; 23% of flies showed highest density of trypanosomes in the distal tip and mid region (20 of 86, Table 1). Thus the view that trypanosome colonisation extends gradually from the proximal end of the proboscis is not completely borne out by the data.

**Table 1:** Trypanosome infection in proboscis. Results for 86 flies infected with *Trypanosoma congolense* savannah (results from strains Gam2, WG81 and 1/148 combined)

Highest density of trypanosomes^a^	No.	%
1. Distal tip	8	9
2. Mid region	7	8
3. Proximal region	41	48
Equal density regions 1 and 2	5	6
Equal density regions 1 and 3	1	1
Equal density regions 2 and 3	15	17
Equal density regions 1, 2 and 3	9	10
Total	86	

^a^Each labrum was scored for density of infection in three regions of approximately equal length excluding the bulb: 1, Distal tip; 2, Mid-region; 3, Proximal region closest to the bulb

All Salivarian trypanosomes, whether they develop in the proboscis or the salivary glands, invade the salivary duct of the hypopharynx, but how they do this is unknown. From the food canal, the only way into the hypopharynx is via its narrow distal end; there is no evidence that trypanosomes penetrate through the wall of the hypopharynx. Whether trypanosomes find the narrow opening of the salivary duct by chance, or by chemoattraction, for example by sensing the concentration gradient of saliva, is unknown.

For *T. congolense*, terminal differentiation to metacyclics occurs in the salivary duct of the hypopharynx, and metacyclics as well as various developmental stages are visible inside the lumen, including attached and unattached forms of various lengths (Additional file 5: Movie 5). Although these trypanosomes move vigorously in situ, there appears to be little movement along the length of the hypopharynx (Additional file 5: Movie 5). It is unclear what barriers prevent *T. congolense* from migrating further like *T. brucei*, and invading the upper reaches of the salivary canal and salivary glands; there is evidently no physical barrier present that restricts the movement of trypanosomes.

## Conclusion

We highlight the remarkable microarchitecture of the tsetse proboscis, in particular the previously undescribed intricate structure of the distal end of the hypopharynx.

## Additional files

Additional file 1: Movie 1. Distal tip of hypopharynx. Altering focal plane to show 3D structure. (ZIP 879 kb)

Additional file 2: Movie 2. Clusters of *Trypanosoma congolense* within the labrum. (ZIP 420 kb)

Additional file 3: Movie 3. Hypopharynx infected with *Trypanosoma congolense*. (ZIP 149 kb)

Additional file 4: Movie 4. Distal tip of hypopharynx infected with *Trypanosoma congolense*. (ZIP 1229 kb)

Additional file 5: Movie 5. Hypopharynx infected with *Trypanosoma congolense*. (ZIP 5572 kb)

## Abbreviations

CM: Cunningham’s medium
PBS: Phosphate buffered saline
SEM: Scanning electron microscopy
